# A sample size planning approach that considers both statistical significance and clinical significance

**DOI:** 10.1186/s13063-015-0727-9

**Published:** 2015-05-12

**Authors:** Bin Jia, Henry S Lynn

**Affiliations:** Department of Biostatistics, School of Public Health, Key Laboratory on Public Health Safety of the Ministry of Education, Fudan University, 130 Dong’an Road, Shanghai, 200032 China

**Keywords:** clinical significance, confidence interval, sample size

## Abstract

**Background:**

The CONSORT statement requires clinical trials to report confidence intervals, which help to assess the precision and clinical importance of the treatment effect. Conventional sample size calculations for clinical trials, however, only consider issues of statistical significance (that is, significance level and power).

**Method:**

A more consistent approach is proposed whereby sample size planning also incorporates information on clinical significance as indicated by the boundaries of the confidence limits of the treatment effect.

**Results:**

The probabilities of declaring a “definitive-positive” or “definitive-negative” result (as defined by Guyatt *et al*., CMAJ 152(2):169-173, 1995) are controlled by calculating the sample size such that the lower confidence limit under *H*_1_ and the upper confidence limit under *H*_0_ are bounded by relevant cut-offs. Adjustments to the traditional sample size can be directly derived for the comparison of two normally distributed means in a test of nonequality, while simulations are used to estimate the sample size for evaluating the hazards ratio in a proportional-hazards model.

**Conclusions:**

This sample size planning approach allows for an assessment of the potential clinical importance and precision of the treatment effect in a clinical trial in addition to considerations of statistical power and type I error.

## Background

The importance of confidence intervals is clearly attested by journal guidelines [1-3] as they “convey information about magnitude and precision of effect simultaneously, and keep these two aspects of measurements closely linked” [4]. For clinical trials, the CONSORT statement [5] stipulates the reporting of the “estimated effect size and its precision (such as 95% confidence interval)” and “how sample size was determined,” but traditional sample size calculations for testing scientific hypotheses consider only statistical significance and power. The precision and clinical importance of the effect that can be depicted by confidence intervals is ignored. Under the usual practice, one calculates the sample size needed to declare some “clinically important difference” statistically significant at the α-level with 1 - β probability. The problem is that there is substantial subjectivity in quantifying this difference, and this can turn the sample size calculation into a moot exercise for choosing a difference to justify the number of patients the study can afford [6]. Frequently, the selected difference ends up larger than what is usual, and thus many studies may display large differences but lack the precision to make them statistically significant. Such shortcomings have led some to argue for reform of current sample size conventions in order to avoid misinterpretation of completed studies and harm to scientific research [7].

What would be helpful is a sample size estimation procedure that provides information on the confidence interval to supply users with information on the clinical significance and precision of the treatment effect in addition to power and statistical significance. Beal [8] suggested selecting sample size such that there is a high probability of the half-width of the confidence interval being less than some prescribed length, conditional on the interval containing the parameter of interest. Similarly, Liu [9] chose the sample size to yield a short confidence interval width but conditional on the rejection of the null hypothesis *H*_0_. Jiroutek *et al*. [10] combined the two by considering the probability of attaining a certain interval width conditional on both rejection of *H*_0_ and inclusion of the true parameter. Cesana *et al*. [11,12] introduced a two-step procedure by first obtaining the sample size according to power and then iteratively increasing the sample size until the probability of obtaining confidence intervals with widths less than the expected interval width under *H*_1_ exceeds a specified level.

In the above methods, the user either has to designate an interval length as reference or rely on the expected interval width, which may not be clinically relevant. A more straightforward alternative is to calculate a sample size such that the confidence limits of the parameter will be bounded by designated cut-offs. Specifically, the sample size is chosen such that according to the confidence limits the result can be deemed “definitive-positive” if there is indeed an effect or deemed “definitive-negative” if there is none. According to Guyatt *et al*. [13], a “definitive-positive” result implies that the lower confidence limit (*LCL*) of the parameter is not only larger than zero, implying a “positive” and statistically significant study, but above a relevant nonzero threshold. Conversely, a “definitive-negative” result implies that the upper confidence limit (*UCL*) is below some nonzero threshold. In hypothesis testing, one does not know whether *H*_1_ or *H*_0_ is true and can only control the probabilities of making a false positive or false negative error. Likewise, in this approach, we control the probabilities of declaring a “definitive-positive” or “definitive-negative” result by calculating the sample size such that *LCL* under *H*_1_ and *UCL* under *H*_0_ are bounded by fixed cut-offs. The following section demonstrates these concepts first for continuous normally distributed data and then for time-to-event data.

## Methods

### Normally distributed data

Consider a randomized 1:1 clinical trial comparing the mean responses between the treatment and control groups. When the response (or appropriately transformed response) can be regarded as normally distributed, the assessment of the treatment effect can be formulated as a hypothesis test of *H*_0_: μ_1_ - μ_0_ = 0 versus *H*_1_: μ_1_ - μ_0_ ≠ 0. The sample size is then given by

1 $$ n=\frac{\sigma^2{\left({Z}_{1-\alpha /2}+{Z}_{1-\beta}\right)}^2}{\delta^2}, $$

where *Z*_γ_ is the γth quantile of the standard normal distribution, (μ_0,_σ_0_) and (μ_1,_σ_1_) are the means and standard deviations of the control and treatment groups, respectively, ${\sigma}^2={\sigma}_0^2+{\sigma}_1^2$, and δ = μ_1_- μ_0_ is the clinically important difference to be detected at level α with power 1 - β.

We first examine how likely the above sample size will yield a “definitive-negative” or “definitive-positive” result by calculating, respectively, the probabilities Pr(*UCL* < *k*_0_δ | *H*_0_) and Pr(*LCL* > *k*_1_δ | *H*_1_) for *k*_0_, *k*_1_ ∈ [0,1]. Without loss of generality, assume δ > 0 and let $\overline{D}$ be the sample estimate of the treatment difference. If σ is known, then

2 $$ \begin{array}{l} \Pr \left(UCL<{k}_0\delta \Big|{H}_0\right)= \Pr \left(\overline{D}+{Z}_{1-\alpha /2}\frac{\sigma }{\sqrt{n}}<{k}_0\delta \Big|{H}_0\right)= \Pr \left(Z<{k}_0\delta \frac{\sqrt{n}}{\sigma }-{Z}_{1-\alpha /2}\right)\\ {}\begin{array}{cccc}\hfill \begin{array}{cc}\hfill \begin{array}{cc}\hfill \begin{array}{cc}\hfill \hfill & \hfill \hfill \end{array}\hfill & \hfill \hfill \end{array}\hfill & \hfill \hfill \end{array}\hfill & \hfill \hfill & \hfill \hfill & \hfill = \Pr \left(Z<\left({k}_0-1\right){Z}_{1-\alpha /2}+{k}_0{Z}_{1-\beta}\right),\hfill \end{array}\mathrm{and}\end{array} $$

3 $$ \begin{array}{l} \Pr \left(LCL>{k}_1\delta \Big|{H}_1\right)= \Pr \left(\overline{D}-{Z}_{1-\alpha /2}\frac{\sigma }{\sqrt{n}}>{k}_1\delta \Big|{H}_1\right)= \Pr \left(Z>\left({k}_1\delta -\delta \right)\frac{\sqrt{n}}{\sigma }+{Z}_{1-\alpha /2}\right)\\ {}\begin{array}{cccc}\hfill \begin{array}{cc}\hfill \begin{array}{cc}\hfill \begin{array}{cc}\hfill \hfill & \hfill \hfill \end{array}\hfill & \hfill \hfill \end{array}\hfill & \hfill \hfill \end{array}\hfill & \hfill \hfill & \hfill \hfill & \hfill = \Pr \left(Z>\left({k}_1-1\right){Z}_{1-\beta }+{k}_1{Z}_{1-\alpha /2}\right),\hfill \end{array}\end{array} $$

where *Z* is the standard normal variable. As *k*_0_, *k*_1_ vary from 0 to 1, these two probability functions are mirror images about 1/2, with Pr(*LCL* > δ /2 | *H*_1_) = Pr(*UCL* < δ /2 | *H*_0_). At the boundaries of 0 and 1, Pr(*LCL* > 0 | *H*_1_) = Pr(*UCL* < δ | *H*_0_) = 1 - β.

Based on the derivations of equations (2) and (3), it can be shown that if the sample size is increased to ${n}_0=n/{k}_0^2$ then Pr(*UCL* < *k*_0_δ | *H*_0_) = 1 - β for *k*_0_ ∈ (0,1) and if it is increased to *n*_1_ = *n*/(1 − *k*_1_)^2^ then Pr(*LCL* > *k*_1_δ | *H*_1_) = 1 - β for *k*_1_ ∈ (0,1). For example, with *k*_0_ = *k*_1_ = 1/2 and sample size *n*_0_ = *n*_1_ = 4*n* both Pr(*LCL* > δ /2 | *H*_1_) = Pr(*UCL* < δ /2 | *H*_0_) = 1 - β. Note that if *k*_0_ = *k*_1_ < 1/2 then *n*_0_ > *n*_1_ and a larger sample size is required to establish a “definitive-negative” compared to a “definitive-positive” result. Conversely, if *k*_0_ = *k*_1_ > 1/2, then *n*_0_ < *n*_1,_ and a larger sample size is needed to establish a “definitive-positive” result. In general, if

4 $$ {k}_0=1-{k}_1\mathrm{and}{n}_0={n}_1=n/{k}_0^2, $$

then Pr(*UCL* < *k*_0_δ | *H*_0_) = Pr(*LCL* > *k*_1_δ | *H*_1_) = 1 - β. For example, if *k*_0_ = 2/3, *k*_1_ = 1/3 and *n*_0_ = *n*_1_ = 9*n* /4 then Pr(*LCL* > δ /3 | *H*_1_) = Pr(*UCL* < 2δ /3 | *H*_0_) = 1 - β.

### Time-to-event data

We extend our proposed method to include time-to-event data, and use this case to show how a simulation-based approach can be used to estimate the sample size when the validity of normal approximation may be in doubt. In situations where a closed-form sample size formula is not readily available or difficult to derive, simulation provides an alternative and offers greater flexibility for adapting to more complicated analyses. Briefly, the initial sample size required to detect the clinically important difference δ at power 1 - β is first calculated and then iteratively increased until Pr(*LCL* > *k*_1_δ | *H*_1_) and Pr(*UCL* < *k*_0_δ | *H*_0_) reach desired levels. The hazard ratio Δ is chosen as the parameter of interest with its corresponding confidence limits *LCL* and *UCL* being estimated using Cox regression. In the following description, we select for simplicity and convenience a single common cut-off by letting *k*_0_ = *k*_1_ = 1/2.

Under the proportional hazards assumption, the initial total sample size *N*_0_ for detecting δ = log_e_Δ at level α and power 1 - β can be estimated using Schoenfeld’s [14] formula,

5 $$ {N}_0=\frac{{\left({Z}_{1-\alpha /2}+{Z}_{1-\beta}\right)}^2}{P_0{P}_1{\left({ \log}_e\Delta \right)}^2}\frac{1}{1-{\pi}_c}, $$

where π_*c*_ is the overall censoring proportion, and *P*_0_ and *P*_1_ are the proportion of subjects in the treatment and control groups, respectively. (Another choice is to use Freedman’s [15] formula, which gives a slightly smaller sample size.)

Time-to-event data are simulated from the exponential distribution since it is most widely used to model time-to-event data under the proportional hazards assumption. Specifically, we simulate exponential survival times *T*_*i*_ and exponential censoring times *L*_*i*_ for subjects *i* = 1, …, *N*_0_/2 in each group, and consider a subject censored whenever *T*_*i*_ < *L*_*i*_. According to Halabi and Bahadur [16], the parameters for the survival and censoring time distributions are given by

6 $$ 2{\pi}_c=\frac{\lambda_c}{\left({\lambda}_0+{\lambda}_c\right)}+\frac{\lambda_c}{\left({\lambda}_1+{\lambda}_c\right)}, $$

where λ_0_, λ_1_ are the hazard rates of the exponential survival times for the control and treatment groups, respectively, and λ_*c*_ is the hazard rate for the exponential censoring time. When π_*c*_ = 0.5, equation (6) reduces to the simple relationship

7 $$ {\lambda}_c=\sqrt{\lambda_0{\lambda}_1}. $$

We set λ_0_ = 1 and select four values, (1.25, 1.5, 1.75, 2.0), for the hazard ratio Δ ≡ λ_1_/λ_0_ = λ_1_. For each value of Δ, the procedure goes through the following steps:

1. With α = 0.05, β = 0.2, *P*_0_ = *P*_1_ = 0.5, π_*c*_ = 0.5, and δ = log_e_(Δ), calculate the initial total sample size *N*_0_ using (5);
2. Simulate *N*_0_/2 independent samples of exponential survival and censoring times for the treatment and control groups with corresponding parameters λ_0_ = 1, λ_1_, and ${\lambda}_c=\sqrt{\lambda_1};$
3. Compare the survival times between the treatment and control groups using Cox regression and compute the 95% confidence interval for log_e_(Δ);
4. Repeat steps (2) and (3) for 10,000 iterations and estimate Pr(*LCL* > δ /2 | *H*_1_) using the proportion of iterations where *LCL* > δ /2;
5. Set Δ = 1 and repeat steps (2) and (3) 10,000 times to estimate Pr(*UCL* < δ /2 | *H*_0_) using the proportion of times when *UCL* < δ /2;
6. Replace *N*_0_ with a larger sample size and repeat steps (2) through (5) until the estimates for both Pr(*LCL* > δ /2 | *H*_1_) and Pr(*UCL* < δ /2 | *H*_0_) are greater than some desired level (for example, 0.8).

The above procedure was programmed using SAS 9.2, and a sample SAS program is provided in the Appendix as reference.

## Results

**Figure 1:**
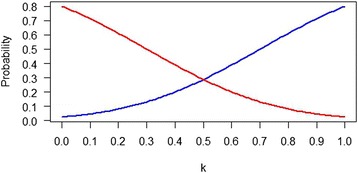
Plot of Pr(*LCL* > *k*δ | *H*_1_) (red curve) and Pr(*UCL* < *k*δ | *H*_0_) (blue curve) for *k* ∈ [0,1], α = 0.05, β = 0.80 in a comparison of normally distributed mean responses with known σ between treatment and control groups for a 1:1 randomized clinical trial.

For comparing the means of normally distributed outcomes, Figure 1 shows that when α = 0.05 and power = 0.8, Pr(*LCL* > *k*δ | *H*_1_) decreases steadily from 0.8 to 0.025 while Pr(*UCL* < *k*δ | *H*_0_) increases steadily from 0.025 to 0.80 as *k* varies from 0 to 1. In fact, these two probability functions are mirror images about *k* = 1/2, where they both equal 0.288. This implies that a trial designed to detect a clinically important difference δ at the 5% significance level with 80% power will be “definitive-positive” about 29% of the time if one wants to say with 95% confidence that the treatment effect must be at least δ /2.

For time-to-event data, the initial total sample size (*N*_0_ = 1264) for detecting a hazard ratio Δ = 1.25 is almost 5/(1 - π_*c*_) or ten times larger than that (*N*_0_ = 132) for detecting Δ = 2.00 according to Schoenfeld’s [14] formula. At these initial sample sizes, the estimates of Pr(*LCL* > 0 | *H*_1_) ranged from 0.79 to 0.81 as expected, while Pr(*UCL* < δ | *H*_0_) ranged from 0.70 to 0.77, slightly less than 0.8. Similarly, estimates for Pr(*LCL* > δ /2 | *H*_1_) ranged from 0.27 to 0.29, close to what is expected for normally distributed data, while estimates of Pr(*UCL* < δ /2 | *H*_0_) are slightly lower than expected, ranging from 0.23 to 0.27. For a specific example, say Δ = 1.75, then *N*_0_ = 204 according to (5) and the estimates of α and β are 0.0485 and 0.2044, respectively. The β estimate implies that 79.6% of the samples have *LCL* > 0 under *H*_1_. But the mean *LCL* is 0.16, thus as shown in Table 1 only 27.7% of the samples have *LCL* > δ /2 = log_e_(1.75)/2 = 0.28. Correspondingly, 95.2% of the samples under *H*_0_ have confidence intervals that include zero, but since the mean *UCL* is 0.42 only 25.4% of the samples have *UCL* < 0.28.

**Table 1:** **Clinical significance and precision of the log-hazard ratio according to the initial and final sample sizes**

Δ	**log**_**e**_ **(**Δ**)**	^**b**^ **λ**_***c***_	***N***		**Pr(** ***LCL*** **>** **δ** **/2 |** ***H***_**1**_ **)**	^**e**^ **CIW**_**1**_	**Pr(** ***UCL*** **<** **δ** **/2 |** ***H***_**0**_ **)**	^**d**^ **CIW**_**0**_
1.25	0.22	1.12	^a^Initial	1264	0.2925	0.322	0.2651	0.314
			^c^Final	5402	0.8241	0.155	0.8016	0.151
1.50	0.41	1.22	^a^Initial	384	0.2759	0.602	0.2658	0.577
			^c^Final	1694	0.8349	0.285	0.8039	0.273
1.75	0.56	1.32	^a^Initial	204	0.2766	0.850	0.2536	0.804
			^c^Final	938	0.8496	0.392	0.8021	0.371
2.00	0.69	1.41	^a^Initial	132	0.2700	1.087	0.2344	1.018
			^c^Final	632	0.8503	0.487	0.8052	0.457

The ^a^initial *N* calculated using equation (5), Schoenfeld’s [14] formula, is the total sample size required to detect a hazard ratio Δ at the 5% level with 80% power, assuming equal subject allocation and a 0.5 overall censoring proportion. ^b^ λ_*c*_ is the hazard rate for the exponential censoring time given by equation (7), and δ. = log_e_(Δ). The ^c^final *N* is the total sample size such that both Pr(*LCL* > δ /2 | *H*_1_) and Pr(*UCL* < δ /2 | *H*_0_) are at least 0.8 as estimated by the proportion of times *LCL* and *UCL* are bounded by δ /2 in 10,000 iterations. ^d^CIW_0_ and ^e^CIW_1_ are the mean width of the 95% confidence intervals under *H*_0_ and *H*_1_, respectively.

Table 1 suggests that sample sizes need to be larger by four to five times the initial sample size before estimates of both Pr(*LCL* > δ /2 | *H*_1_) and Pr(*UCL* < δ /2 | *H*_0_) are above 0.8. For example, with Δ = 1.75, the mean *LCL* for samples under *H*_1_ equals 0.38 when the sample size reaches 938 (4.6 times *N*_0_), and 85.0% of the samples then have *LCL* > δ /2 = 0.28. In addition, at this sample size, the mean *UCL* for samples under *H*_0_ equals 0.19, and 80.2% of the samples have *UCL* < 0.28. In terms of confidence interval width, the final sample sizes yield confidence interval widths that are between 0.4 to 0.5 times narrower than those at the initial sample sizes. For example, with Δ = 1.75 and a final sample size of 938, the mean confidence interval widths are 0.37 and 0.39 under *H*_0_ and *H*_1_, respectively, and 0.46 times narrower than the corresponding mean confidence interval widths at the initial sample size of 204.

## Discussion

Many researchers realize that a traditional sample size calculation for testing *H*_0_: μ_1_ - μ_0_ = 0 versus *H*_1_: μ_1_ - μ_0_ ≠ 0 with α = 0.05 and 80% power to detect a clinically important difference δ implies that: 1) 95% of its 95% confidence intervals for μ_1_ - μ_0_ will include zero when *H*_0_ is true, and 2) 80% of the 95% confidence intervals will exclude zero when *H*_1_ (that is, μ_1_ - μ_0_ = δ) is true. However, a confidence interval with a *LCL* that is barely larger than zero may indicate a statistically significant treatment effect but be unconvincing to investigators who desire a “definitive-positive” result [13]. In contrast, a confidence interval that includes zero and demonstrates a “statistically nonsignificant” effect may be more convincing as a “definitive-negative” result when its *UCL* is small. Therefore, we propose that information on Pr(*LCL* > cut-off | *H*_1_) and Pr(*UCL* < cut-off | *H*_0_) be available to assist investigators in gauging the clinical significance of the treatment effect. For example, a plot similar to Figure 1 can be provided as a supplement to the usual sample size calculation or the investigator can directly estimate the sample size required such that *LCL* and *UCL* are bounded by relevant cut-offs with high probability. This offers a more consistent approach since the confidence interval becomes an important component in the design of clinical trials and not solely for analysis.

One question for this method concerns how a clinically relevant cut-off can be selected. Since δ, the clinically important difference, is already defined in the original sample size calculation, a convenient choice is to specify the cut-off with respect to δ. Given the uncertainty involved in quantifying δ and the tendency to inflate it [6], we set the cut-off equal to *k*δ for *k* ∈ (0,1). This bypasses the need to additionally specify a confidence interval reference width [8-10] or calculate an expected confidence interval width [11,12]. For example, δ /2 can be used as the cut-off since it gives equal consideration to the expected precision of symmetrical intervals under *H*_0_ and *H*_1_. However, it should be stressed that there is no requirement for intervals under *H*_0_ and *H*_1_ to be given equal emphasis or for the boundaries of *LCL* and *UCL* to be the same. A researcher may well choose different cut-offs corresponding to a “definitive-positive” and a “definitive-negative” result; for example, *LCL* > 3δ /4 and *UCL* < δ /4 or *LCL* > δ /3 and *UCL* < 2δ /3.

Previous considerations of sample size estimation by controlling statistical power and precision often involve complex calculations even for normally distributed or binary outcomes. The current proposal is pedagogically straightforward as it simply focuses on the position of the confidence limits in relation to clinically relevant boundaries. Greenland [17] designed a method that provides high power to discriminate between the parameter values under *H*_0_ and *H*_1_. A sample size was chosen such that the discriminatory power, min{ Pr(*LCL* > 0 | *H*_1_), Pr(*UCL* < δ | *H*_0_)}, equals a specified level. Our method also focuses on the probabilities of the lower and upper confidence limits being bounded, but the boundaries are different as Greenland was not thinking of clinically important effect sizes but the original parameter values under *H*_0_ and *H*_1_.

The condition *LCL* > *k*_1_δ corresponds to the alternative hypothesis for a superiority test of *H*_0_: μ_1_ - μ_0_ ≤ *k*_1_δ versus *H*_1_: μ_1_ - μ_0_ > *k*_1_δ. However, the sample size *n*_1_ to attain a “definitive-positive” result is different from the sample size for the superiority test since the former is two-sided while the latter is one-sided. For example, with α = 0.05, β = 0.2, σ^2^ = 2, δ = 1, and *k*_1_ = 1/2, equations (1) and (4) imply that *n*_1_ = 4×16 = 64, while the sample size for the superiority test, as given by

$$ \frac{\sigma^2{\left({Z}_{1-\alpha }+{Z}_{1-\beta}\right)}^2}{{\left(\delta -{k}_1\delta \right)}^2}, $$

equals 50. More importantly, our method calculates not only the sample size involving *LCL* > *k*_1_δ but also that for *UCL* < *k*_0_δ.

## Conclusions

In summary, our proposed method allows the researcher to calculate the sample size for a clinical trial not only according to the specifications of statistical significance (that is, α and β) but also in terms of clinical significance as judged by the boundaries of the confidence limits. For normally distributed data, simple formulae are available and their results serve as a reference for sample size planning when analyzing other types of data. For example, to ensure that *LCL* and *UCL* are both bounded by δ /2 the sample size needs to be increased 4-fold when comparing normally distributed means. Likewise, when evaluating the hazard ratio for time-to-event data, simulation results also suggest that sample sizes need to be 4 to 5 times larger. The results of our method indicate that sample size needs to be increased but our intention is not to mandate larger sample sizes per se. Such an effort may be futile since in practice cost constraints force clinical trials to aim for the smallest possible sample size What is important is that researchers be informed, for example by a graph similar to Figure 1, as to how their sample size will affect judgments of clinical significance using confidence intervals. In this respect, our proposal directs attention back to the importance of gauging effect sizes using confidence intervals, and is consistent with the predicted confidence intervals Goodman and Berlin [6] advocated to help investigators better understand the idea of statistical power when calculating sample size.

## Appendix

Sample SAS program to estimate the total sample size for testing *H*_0_: Δ = 1 versus *H*_1_: Δ ≠ 1 such that Pr(*LCL* > δ /2 | *H*_1_) = Pr(*UCL* < δ /2 | *H*_0_) = 1 - β. Survival and censoring times are assumed to be exponentially distributed, and the overall censoring proportion equals 0.5. The initial sample size is estimated using Schoenfeld’s [14] formula for detecting δ = log_e_(Δ) with 80% power at the 5% significance level.

## Abbreviations

CONSORT: Consolidated Standards of Reporting Trials
LCL: lower confidence limit
UCL: upper confidence limit
